# Achievement Goal Profiles and Academic Performance in Mathematics and Literacy: A Person-Centered Approach in Third Grade Students

**DOI:** 10.3390/jintelligence13090108

**Published:** 2025-08-27

**Authors:** Justine Fiévé, Maxim Likhanov, Pascale Colé, Isabelle Régner

**Affiliations:** Centre de Recherche en Psychologie et Neurosciences, Aix Marseille University, CNRS, CRPN, 13003 Marseille, France

**Keywords:** achievement goal profiles, gender differences, mathematics performance, literacy performance, latent profile analysis, elementary school

## Abstract

In spite of the ever-growing body of research in achievement goal profiles and their contribution to performance, the research on young children is quite limited. This study examined achievement goal profiles related to mathematics and literacy performance among third-grade students (*N* = 185, *M* = 8.73 years; 98 girls), using Latent Profile Analysis. Four distinct profiles emerged—*Mastery-Oriented*, *Approach-Oriented*, *High Multiple-Goals*, and *Moderate Multiple-Goals*—that were highly similar across math and literacy (*contingency coefficient* = 0.59). Schoolchildren endorsing the *Approach-Oriented* profile demonstrated higher achievement compared to those with *High Multiple-Goals* or *Moderate Multiple-Goals* profiles, which involved more avoidance goals and were less adaptive (with up to 8% of variance explained by profile). Gender differences were observed: girls were more likely to endorse profiles combining multiple goals, whereas boys more often endorsed mastery or approach profiles. These results highlight early inter-individual differences in motivational development, observable in both mathematics and literacy. Promoting adaptive goal profiles in early education may enhance academic engagement and help reduce emerging motivational disparities.

## 1. Introduction

Within the achievement goal literature, increasing attention has been paid to the benefits of adopting a person-centered approach to better understand how students’ motivational patterns relate to academic outcomes. Rather than examining achievement goals in isolation, person-centered approaches focus on how multiple goals co-occur within individuals, offering a nuanced perspective on the complex motivational configurations that may influence learning and performance (Hornstra et al. 2017; Zhang et al. 2023); however, research in this direction is yet scarce. Further, while extensive research has investigated achievement goals in secondary and post-secondary education, relatively little is known about how such motivational profiles relate to academic outcomes in primary school settings (Schwinger et al. 2016; Wormington and Linnenbrink-Garcia 2017). The present study seeks to address these gaps by identifying achievement goal profiles among third-grade students, exploring gender differences in profile membership, and examining their associations with academic performance in mathematics and literacy.

### 1.1. Achievement Goals Framework

The achievement goal theory has undergone several conceptual refinements since its inception. Initially, researchers distinguished between two primary orientations: mastery goals, centered on learning and understanding, and performance goals, focused on demonstrating ability relative to others (Dweck and Leggett 1988; Nicholls 1984). This binary framework later evolved into a trichotomous model that differentiated between performance-approach goals (striving to outperform others) and performance-avoidance goals (aiming to avoid doing worse than others; Elliot and Harackiewicz 1996) in addition to mastery. The most widely adopted extension, the 2 × 2 model proposed by Elliot and McGregor (2001), introduced a further distinction within mastery goals, between mastery-approach (striving to learn and develop competence) and mastery-avoidance goals (avoiding misunderstanding and failure to master a task).

This expanded framework has significantly advanced our understanding of students’ academic motivation by capturing both the valence (approach vs. avoidance) and the orientation (mastery vs. performance) of students’ goals. However, empirical research, particularly with younger children, has not always reflected this theoretical breadth. In many studies, especially those conducted in elementary school settings, mastery-avoidance goals are omitted. While this is often attributed to the difficulty of assessing mastery-avoidance in young populations due to the cognitive complexity of the items involved (Carr and Marzouq 2011), such exclusions also reflect researchers’ a priori theoretical and methodological decisions. These decisions are not necessarily based on students’ actual capacity to differentiate between goal types but rather on assumptions regarding the developmental appropriateness of certain constructs. For example, the inclusion of a valence-based distinction in the case of mastery goals was motivated by theoretical considerations, rather than strong empirical support. Consequently, the empirical relevance of mastery-avoidance goals has been debated, with some questioning their validity (Pintrich 2003; Sideridis and Mouratidis 2008). However, recent findings suggest that even fifth and sixth graders are capable of distinguishing among all four goals defined in the 2 × 2 model (Charalampous 2018), calling for more comprehensive investigations of achievement goals in younger learners.

### 1.2. Emergence of Achievement Goal Profiles

While the 2 × 2 model has deepened our understanding of motivational orientations, it is now widely recognized that students rarely pursue achievement goals in isolation. Rather, they tend to endorse multiple goals simultaneously, combining them in diverse ways depending on contextual and individual factors (Hornstra et al. 2017; Schwinger and Wild 2012). This recognition has given rise to the concept of achievement goal profiles, patterns that reflect students’ complex motivational orientations in real learning environments.

Thus, a central question in achievement goal research concerns which combinations, or profiles, of goals are most conducive to academic success. From a traditional mastery goal perspective, a substantial body of research has confirmed a generally positive association between mastery-approach goals and academic performance, although the strength of this relationship can vary depending on contextual and individual factors (e.g., Alhadabi and Karpinski 2019; An et al. 2022; Darnon et al. 2018; Guo et al. 2023; Phan 2010). Mastery-approach goals are considered particularly adaptive, as they are associated with the use of deep learning strategies, enhanced intrinsic motivation, and greater effort, perseverance, and progress toward academic objectives, which in turn support optimal performance (Kaplan et al. 2002; Katz-Vago and Benita 2024; Midgley et al. 2001). Conversely, performance-avoidance goals have been consistently linked to negative academic and psychological outcomes. These goals have been linked to lower academic achievement, reduced well-being, diminished self-efficacy, and increased levels of anxiety and depressive symptoms (e.g., An et al. 2022; Bruno et al. 2019; Yu et al. 2023). Students who endorse performance-avoidance goals tend to avoid seeking help, fear being perceived as incompetent, and demonstrate increased anxiety and vulnerability to depression (e.g., Middleton and Midgley 1997; Sideridis 2005).

Findings regarding performance-approach goals are more equivocal. Studies report positive (Alhadabi and Karpinski 2019; Senko and Dawson 2017; Wirthwein and Steinmayr 2021), negative (Katz-Vago and Benita 2024; Senko and Dawson 2017), or null (Dompnier et al. 2013; Senko and Dawson 2017; Wirthwein and Steinmayr 2021) associations between these goals and school achievement (for reviews see Harackiewicz et al. 2002b; Hulleman et al. 2010). These mixed results have given rise to divergent theoretical interpretations. Proponents of a multiple goal perspective argue that students may benefit from pursuing both mastery- and performance-approach goals simultaneously (Barron and Harackiewicz 2001; Harackiewicz et al. 2002a, 2002b). According to this view, the co-endorsement of high levels of mastery-approach and performance-approach goals can lead to enhanced engagement and academic performance, particularly in the short term.

However, advocates of the mastery goal perspective emphasize the potential downsides of performance-approach goals, even when combined with mastery goals. While performance-approach goals may initially promote motivation and competitiveness, they may also foster pressure, heighten anxiety, reduce intrinsic interest, and discourage adaptive behaviors such as help-seeking, especially when students are confronted with failure or negative feedback (Chung et al. 2020; Elliot and Church 1997; Harris et al. 2023; Leenknecht et al. 2019; Tuckey et al. 2002). As such, the short-term benefits of performance-approach goals may ultimately be outweighed by their longer-term emotional and behavioral costs.

### 1.3. Achievement Goal Profiles

In recent years, researchers have increasingly turned to person-centered approaches to better understand how students combine different types of achievement goals. Rather than examining each goal in isolation, methods such as Latent Profile Analysis (LPA; Pastor et al. 2007) identify groups of students who tend to endorse similar patterns of goals. Across educational levels from middle school to university, this type of analysis has revealed several recurring motivational profiles. These include, for example, students who are focused mainly on learning and improvement (*Mastery-Oriented*), students who pursue both learning and performance simultaneously (*Approach-Oriented*), and others who show generally low motivation or who are driven by a fear of failure (e.g., *Multiple-Goals*, *Amotivated* or *Avoidance-Oriented*; Bae and DeBusk-Lane 2018; Lee et al. 2020; for a meta-analysis, see Wormington and Linnenbrink-Garcia 2017).

For example, Luo et al. (2011) studied over 1700 high school students and found four main motivational profiles based on the trichotomous model: *Diffuse* (i.e., moderate levels of multiple goals), *Moderate Mastery* (with low performance goals), *Success Oriented* (i.e., moderate mastery with high performance goals), and *Approach* (i.e., high mastery-approach and performance-approach with low performance-avoidance goals). Similarly, Jang and Liu (2012), using the 2 × 2 framework, identified five profiles among middle school students: *High Multiple-Goals*, *High Mastery-Approach*, *Low Multiple-Goals*, *High Mastery-Avoidance*, and *Low Performance*.

However, much less is known about how these motivational profiles develop in younger students, particularly in elementary school. In a meta-analysis, Wormington and Linnenbrink-Garcia (2017) identified only three person-centered studies on achievement goal profiles in primary school settings: Meece and Holt (1993), Tapola et al. (2014) and Veermans and Tapola (2004). These studies differ substantially in their theoretical foundations and measurement approaches, making direct comparisons difficult. For instance, Meece and Holt (1993) conducted a cluster analysis based on early goal types (task-mastery, ego-social, and work-avoidance), which predate the 2 × 2 framework proposed by Elliot and McGregor (2001). Tapola et al. (2014) used latent class analysis to identify five goal types, including mastery-intrinsic and mastery-extrinsic distinctions, while Veermans and Tapola (2004) applied traditional cluster analysis using three goals: learning orientation, performance orientation, and avoidance orientation.

Also, due to the question of the validity of the mastery-avoidance goal among young people, most have relied on a trichotomous model encompassing mastery-approach, performance-approach, and performance-avoidance goals (e.g., Hornstra et al. 2017; Jansen in de Wal et al. 2015; Schwinger et al. 2016; Schwinger and Wild 2012). Although informative, these studies underscore the scarcity of research using the 2 × 2 framework and robust person-centered methods, highlighting the need for more comprehensive investigations of achievement goal profiles in younger populations. Despite the tendency to omit mastery-avoidance goals, studies using the trichotomous framework have consistently identified four motivational profiles in elementary school populations (e.g., Hornstra et al. 2017; Jansen in de Wal et al. 2015; Schwinger et al. 2016; Schwinger and Wild 2012): (1) a *High Multiple-Goals* profile, characterized by strong endorsement of all three goals; (2) a *Moderate Multiple-Goals* profile, showing moderate endorsement across goals; (3) a *Mastery-Oriented* profile, dominated by high mastery-approach goals and low performance goals; (4) an *Approach-Oriented* profile, combining high mastery- and performance-approach goals with low performance-avoidance.

### 1.4. Achievement Goal Profiles and Academic Performance

A growing body of person-centered research has examined how different achievement goal profiles relate to academic outcomes across educational levels (e.g., Kim et al. 2025; Ning 2016; Pastor et al. 2007; Tuominen-Soini et al. 2012; Wormington and Linnenbrink-Garcia 2017). These studies consistently show that certain combinations of goals, particularly those emphasizing mastery-approach, are associated with more adaptive learning behaviors and stronger academic performance. In contrast, profiles involving avoidance goals, or lacking strong mastery orientation, are more likely to predict lower achievement and less effective self-regulation. In elementary school contexts, Hornstra et al. (2017) found that students endorsing both mastery- and performance-approach goals (while minimizing performance-avoidance tendencies) displayed greater effort in language and mathematics, along with more progress in language achievement over time. These findings suggest that combining mastery and performance-approach orientations can be beneficial at early developmental stages, provided that performance-avoidance goals remain low.

These findings align with research showing that students focused on learning, understanding, and developing competence generally perform better in both standardized assessments and school grades (Zhang et al. 2016, 2023). Longitudinal data further reveal that students who maintain a mastery-oriented profile over time tend to achieve greater academic success, even across the transition to secondary school, likely due to the deep learning strategies and intrinsic motivation associated with mastery goals (Zhang et al. 2023). Conversely, profiles that include high levels of performance-avoidance, such as the *High Multiple-Goal* profile, are more often linked to lower academic outcomes. One possible explanation lies in the association between avoidance motivations, test anxiety, and cognitive interference. For instance, in a longitudinal study spanning Grades 3 to 7, Schwinger and Wild (2012) found that students with a *High Multiple-Goal* profile showed declining math achievement in later grades. Notably, students with initially lower academic performance were more likely to shift into this profile over time, suggesting a reciprocal relationship between achievement and motivational orientation. These findings were corroborated by Zhang et al. (2023), who showed that *Mastery-Oriented* students consistently outperformed their peers one and two years after transitioning to secondary school, whereas *High Multiple-Goal* profile offered no long-term academic benefit.

### 1.5. Achievement Goals Profiles and Gender Differences

Previous research highlighted gender differences in the adoption of achievement goal profiles during elementary school. For example, in a large-scale latent profile analysis involving 4387 fourth-grade German students, Zhang et al. (2016) found a significant association between gender and motivational profile membership. Specifically, 38% of girls were assigned to a *Mastery-Oriented* profile compared to 29.6% of boys, whereas 45.6% of boys, versus 33.5% of girls, were classified in a *High Multiple-Goal* profile, characterized by high endorsement of both mastery and performance goals. These findings are consistent with previous profile research (Luo et al. 2011; Roeser et al. 1996; Schwinger and Wild 2012). These gender-related patterns in profile membership were replicated in a longitudinal follow-up study by Zhang et al. (2023), conducted with a different sample of 1764 students from grades 4 to 7. A significant correlation was found between gender and latent class membership (*r* = 0.142, *p* < .001). In this study, 40.4% of girls (compared to 31.0% of boys) were assigned to a *Mastery-Oriented* profile, while 43.3% of boys (versus 29.8% of girls) were classified in the *High Multiple-Goal* profile.

Beyond profile prevalence, gender differences have also been observed in the structural relations between achievement goals. Meece et al. (2003), for instance, reported a stronger correlation between mastery and performance goals in boys (*r* = 0.46) than in girls (*r* = 0.28), suggesting that boys are more likely to pursue multiple goals simultaneously. Similarly, Hulleman et al. (2010) identified gender as a significant moderator of the correlation between mastery and performance-approach goals: the correlation was substantial in all-male samples (*r* = 0.39) but nearly absent in all-female samples (*r* = 0.02).

Schwinger et al. (2016) provided further insights into the mechanisms underlying these differences by examining how gender interacts with motivational beliefs and self-perceptions. Based on longitudinal data from 542 students followed from third to fourth grade, their analyses revealed that girls were significantly more likely than boys to belong primarily to *Mastery-Oriented* profile. Additionally, students with higher academic self-concept at the end of fourth grade were more likely to adopt a *Mastery-Oriented* profile compared to those in *High Multiple-Goal* (*OR* = 0.39) or *Moderate Multiple-Goal* profiles (*OR* = 0.26). The study also showed that boys who endorsed an entity theory of intelligence (i.e., refers to the belief that intelligence is a fixed, innate quality, unlikely to change through effort or learning) were particularly unlikely to adopt mastery-focused profiles, highlighting how beliefs about ability may interact with gender to shape goal adoption.

It is worth noting, however, that gender differences are not always consistent across age groups or academic domains. In samples of younger students and in subject-specific contexts such as mathematics, boys and girls have sometimes shown similar levels of mastery goal endorsement, while boys report higher levels of performance-approach and performance-avoidance goals (e.g., Anderman and Midgley 1997; Meece et al. 2003). These findings suggest that motivational profiles are influenced by both developmental and contextual factors, and that gender effects may vary depending on subject matter.

### 1.6. The Current Study

To sum up, research on achievement goal profiles in primary school students remains limited, despite growing recognition of the importance of motivational patterns during early academic development. Existing studies have primarily focused on older students, leaving gaps in our understanding of how goal orientations emerge and function at earlier stages.

The present study addresses this gap by investigating achievement goal profiles among third-grade students using the full 2 × 2 framework (Elliot and McGregor 2001) and examining how these profiles relate to academic performance. Our first objective is to determine whether distinct motivational profiles can be identified at this early stage of schooling. To this end, we apply Latent Profile Analysis (LPA) to examine how students cluster based on their endorsement of mastery-approach, mastery-avoidance, performance-approach, and performance-avoidance goals. Drawing on prior findings on elementary school students (e.g., Wormington and Linnenbrink-Garcia 2017), we expect to identify four profiles: *High Multiple-Goals*, *Moderate Multiple-Goals*, *Mastery-Oriented*, and *Approach-Oriented*. Further, in line with previous research showing gender-related patterns in goal adoption (e.g., Schwinger et al. 2016; Zhang et al. 2016, 2023), we hypothesize that girls will be more likely to belong to the *Mastery-Oriented* profile, whereas boys will be more frequently represented in the *Multiple-Goal* profiles.

Our second objective is to examine whether these profiles are differentially associated with academic achievement in the two core domains of early education: mathematics and literacy. These foundational skills are critical to later academic success and are typically emphasized in third grade curricula. We hypothesize that students in the *Approach-Oriented* profile will exhibit the highest levels of academic achievement. Students in the *Mastery-Oriented* profile are expected to follow, given the well-established benefits of mastery goals. In contrast, students in the *Moderate* or *High Multiple-Goals* profiles may demonstrate lower achievement due to the presence of competing or avoidance-driven goals.

## 2. Materials and Methods

### 2.1. Participants

The present study involved 185 third-grade students (98 girls, 87 boys) aged 8 to 10 years (*M* = 8.73, *SD* = 0.36) from 20 classes across eight public schools in the Bouches-du-Rhône department, France. These schools were selected to ensure socioeconomic diversity and assessed using the Social Position Index (SPI), a standardized continuous measure tailored to the French context (*M* = 100, *SD* = 30; Dauphant et al. 2023; Rocher 2016). This index captures multiple dimensions of social stratification, including parental education, occupational status, material conditions, and cultural capital. In this sample, SPI scores ranged from 54 (less privileged) to 180 (highly privileged), reflecting substantial socioeconomic heterogeneity. This composite index was subsequently used as the measure of socioeconomic status (SES) in the present study. Of the 300 families contacted, 209 provided consent, and 24 students (11%) were excluded due to neurodevelopmental disorders.

### 2.2. Sensitivity Power Analysis

A priori sample size calculation was not feasible due to practical constraints inherent to research in school settings, such as the need for authorization from school principals, parental consent, and compliance with ethical and administrative procedures. Instead, a post hoc sensitivity power analysis was conducted using G*Power (v.3.1.9.7; Faul et al. 2007) for the analysis of covariance (ANCOVA; *N* = 185; two groups: boys and girls; one covariate: SES; *α* = 0.05; *power* = 0.80). This analysis indicated that the smallest detectable effect size was *Cohen’s d* = 0.207, suggesting that the study had adequate power to detect effects of small-to-moderate magnitude related to gender and psychological predictors.

### 2.3. Procedure

The assessments were conducted in small groups of 10 to 16 students, in a quiet room within their school, over the course of a half-day session. Each session began with a two-hour standardized assessment of academic performance in mathematics and literacy, divided into two 1-h sessions with a 30-min break in between. This was followed by a 30-min self-report questionnaire session, which included the achievement goals measure as well as additional measures unrelated to the present study, collected as part of a larger research project. The achievement goals questionnaire was structured in two sections, one for mathematics and the other for literacy (defined for students as French language skills, for clarity). Each item was read aloud by the experimenter, while the five response options were simultaneously displayed using printed facial expression visuals, ranging from “dark red and unhappy” (1 = strongly disagree) to “dark green and happy” (5 = strongly agree). All sessions were conducted under standardized conditions, with trained researchers providing consistent instructions and ensuring timing uniformity across groups.

### 2.4. Measures

*Achievement Goals*. Achievement goals were assessed using the French version (Loose et al. 2012) of the scale originally developed by Elliot and McGregor (2001), which includes 12 items representing four goal orientations. Students completed the scale separately for mathematics and literacy, whose sample items include: mastery-approach (“*In mathematics/French, you want to learn as much as possible*”), mastery-avoidance (“*What worries you in mathematics/French is not understanding the lessons or exercises*”), performance-approach (“*It is important for you to have the best results in mathematics/French in the class*”), and performance-avoidance (“*You want to avoid having worse results in mathematics/French compared to the other students in your class*”) goals.

Confirmatory factor analyses showed a poor fit for the four-factor model, probably due to low factor loadings of items on the performance-avoidance factor. Additionally, the internal consistency of the performance-avoidance subscale was low (mathematics: *α* = 0.43; literacy: *α* = 0.34). Consistent with prior research, the performance-avoidance goal often exhibits the lowest Cronbach’s alpha values among achievement goal subscales (e.g., Agbuga et al. 2015; Cano and Berbén 2009; Darnon and Butera 2005; Elliot and McGregor 2001; Finney et al. 2004; Jowkar et al. 2014; Van Yperen 2006). As a result, this dimension was excluded from subsequent analyses for both mathematics and literacy. Model fit improved significantly after its removal, with acceptable indices observed in both domains (mathematics: *χ*^2^(24) = 35.20, *p* = .066; *CFI* = 0.97; *TLI* = 0.96; *RMSEA* = 0.05; *SRMR* = 0.05; literacy: *χ*^2^(24) = 28.00, *p* = .260; *CFI* = 0.99; *TLI* = 0.99; *RMSEA* = 0.03; *SRMR* = 0.05). The remaining three dimensions showed adequate reliability: mastery-approach (mathematics: *α* = 0.72; literacy: *α* = 0.70), mastery-avoidance (mathematics: *α* = 0.66; literacy: *α* = 0.71), and performance-approach (mathematics: *α* = 0.76; literacy: *α* = 0.82).

*Mathematics and Literacy Performance*. Academic performance in mathematics and literacy was assessed using standardized tests developed in collaboration with classroom teachers. The mathematics test included exercises in number decomposition, arithmetic operations, and problem-solving (four items in each category). The literacy test assessed spelling (two-sentence dictation), written expression (a three-sentence story based on pictures), and reading comprehension (five questions targeting both explicit and implicit information). To ensure uniform testing conditions, time constraints were applied to each item and therefore students were not allowed to return to previous items. All test scores were converted to a 20-point scale to facilitate comparison between mathematics and literacy performance.

### 2.5. Descriptive Statistics and Missing Data Handling

Descriptive statistics, including means, standard deviations, and correlations, for all variables were computed to summarize sample characteristics. Missing data were assumed to be Missing at Random, as confirmed by Little’s test (*χ*^2^(82) = 71.95, *p* = .778), and addressed using Expectation-Maximization estimation (Little 1988). The proportion of missing data was minimal, ranging from 0.54% (mathematics achievement goals and parental academic involvement) to 2.16% (literacy achievement goals), with no variable exceeding the 5% threshold.

### 2.6. Analytical Strategy

*Identification of Achievement Goals Profiles*. To identify achievement goal profiles separately for mathematics and literacy, we used Latent Profile Analysis (LPA), a person-centered statistical approach that classifies individuals into subgroups based on shared patterns of achievement goal orientations (Hornstra et al. 2017; Jansen in de Wal et al. 2015; Muthén and Muthén 2000; Pastor et al. 2007). Separate LPA models were estimated for mathematics and literacy, with model-fit indices including Akaike’s Information Criterion (AIC; Akaike 1998), Bayesian Information Criterion (BIC; Schwarz 1978), sample-size adjusted BIC (SABIC; Sclove 1987) and a significant Bootstrap Likelihood Ratio Test (BLRT; Nylund et al. 2007) that supported a four-profile solution in both domains. A detailed description of the LPA parameters and the step-by-step process for identifying profiles is provided in the Supplementary Materials.

*Gender Distribution in Achievement Goals Profiles*. To examine the relationship between students’ achievement goal profiles across the mathematics and literacy domains, as well as to explore potential differences by gender, we conducted a series of chi-square tests of independence. Gender was coded as 0 = girl and 1 = boy. First, a chi-square test was used to assess the association between students’ profile membership in mathematics and literacy. Then, separate chi-square analyses were conducted for girls and boys to determine whether the alignment of profiles across academic domains differed by gender. These analyses allowed us to investigate the extent to which students maintained consistent motivational profiles across subjects, and whether this consistency varied as a function of gender.

*Associations between Achievement Goal Profiles and Academic Performance*. To examine the associations between achievement goal profiles and academic performance, we conducted separate ANCOVA models for mathematics and literacy. In each analysis, academic performance (mathematics or literacy scores) served as the dependent variable, while gender and achievement goal profiles were included as fixed factors. SES was included as a covariate to control for its potential influence.

## 3. Results

### 3.1. Descriptive Statistics

Descriptive statistics, including means, standard deviations, and Pearson correlations for all variables are presented in Table 1. Gender differences were observed: boys (*M* = 14.18, *SD* = 3.02) outperformed girls in mathematics (*M* = 13.19, *SD* = 3.31; *Cohen’s d* = 0.31, *p* = .046), whereas girls (*M* = 13.28, *SD* = 3.73) outperformed boys in literacy (*M* = 11.32, *SD* = 3.89; *Cohen’s d* = 0.52, *p* < .001).

We also identified gender differences for mastery-avoidance goals in both domains. In mathematics, girls (*M* = 3.45, *SE* = 0.11) reported higher endorsement than boys (*M* = 3.01, *SE* = 0.12; *Cohen’s d* = 0.41, *p* = .006), and a similar pattern was observed in literacy, with girls (*M* = 3.56, *SE* = 0.12) again scoring higher than boys (*M* = 3.10, *SE* = 0.13; *Cohen’s d* = 0.41, *p* = .006). Furthermore, mastery avoidance goals were negatively correlated with domain specific performance in both mathematics (*r* = −0.22, *p* = .003) and literacy (*r* = −0.22, *p* = 0.003), suggesting that girls are more likely than boys to worry about not mastering tasks or misunderstanding. In contrast, no significant gender differences were found for mastery-approach or performance-approach goals. Detailed descriptive statistics including some Pearson correlation graphs are available in the Supplementary Materials.

### 3.2. Achievement Goal Profiles Across Academic Domains

**Figure 1:**
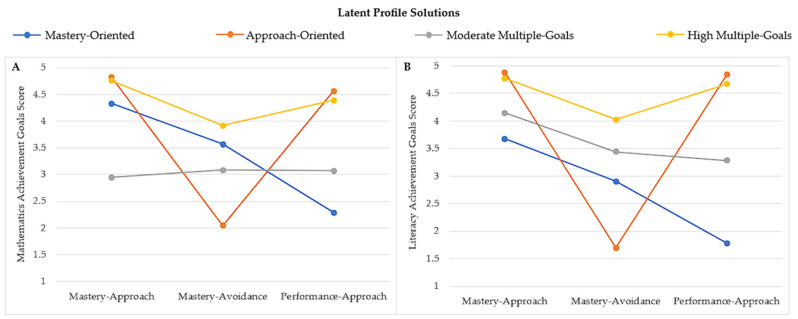
Achievement goal profiles identified through LPA method: (**A**) in mathematics; (**B**) and in literacy.

Figure 1 presents the achievement goal profiles identified in mathematics (Panel A) and literacy (Panel B). LPA revealed the same four motivational patterns in mathematics and literacy: (1) *Mastery-Oriented*, characterized by high mastery-approach and mastery-avoidance goals, with low performance-approach goals; (2) *Approach-Oriented*, combined high mastery-approach and performance-approach goals, with low mastery-avoidance goals; (3) *Moderate Multiple-Goals*, reported moderate levels of the three goals; and (4) *High Multiple-Goals*, marked by high levels of the three goals.

Domain-specific differences nevertheless emerged. The *Mastery-Oriented* profile was nearly twice as prevalent in literacy (12.43%) as in mathematics (7.57%), while the *Approach-Oriented* profile appeared more frequently in mathematics (28.65%) than in literacy (16.76%). The *Moderate Multiple-Goals* profile was more common in literacy (27.03%) compared with mathematics (12.43%). In contrast, the *High Multiple-Goals* profile was the most frequent in both domains, representing 51.35% of students in mathematics and 43.78% in literacy.

### 3.3. Gender Differences in Profile Membership

A chi-square test revealed a significant association between students’ achievement goal profiles across the mathematics and literacy domains (*χ*^2^(9) = 96.10, *p* < .001, *contingency coefficient* = 0.59), indicating a strong association between the two sets of profiles. When analyses were conducted separately by gender, this strong association remained significant for both girls (*χ*^2^(9) = 39.20, *p* < .001, *cc* = 0.53), and boys (*χ*^2^(9) = 56.40, *p* < .001, *cc* = 0.63), suggesting consistent alignment of profile membership across domains within each gender group. Table 2 presents the cross-tabulation of profile membership in mathematics and literacy, allowing for a detailed analysis of students’ profile stability across domains. Overall, students did not systematically maintain the same profile in both subjects, but stability patterns varied depending on the profile type and gender.

Among girls, the highest level of stability was observed in the *High Multiple-Goals* group, with 64.40% retaining the same profile across mathematics and literacy. Stability was moderate in the *Mastery-Oriented* and *Moderate Multiple-Goals* groups, with 50.00% of girls in each of these profiles maintaining their initial orientation. In contrast, girls initially classified as *Approach-Oriented* in mathematics were less stable: only 30.40% retained this profile in literacy, while 43.50% moved to the *High Multiple-Goals* profile, suggesting a shift toward a more complex motivational pattern.

For boys, profile stability was generally higher in the *Mastery-Oriented* group, where 62.50% retained their profile across domains and 37.50% moved to the *Moderate Multiple-Goals* group. Boys in the *Approach-Oriented* group also showed notable consistency, with 56.70% maintaining this profile in literacy, while 30.00% moved to the *High Multiple-Goals* profile and 13.30% to the *Moderate Multiple-Goals* profile. In contrast, stability was lowest among boys initially classified as *Moderate Multiple-Goals*: only 23.10% retained their profile, while 38.50% moved to the *Mastery-Oriented* group and 30.80% to the *High Multiple-Goals* group. Boys in the *High Multiple-Goals* group showed relatively high stability, with 60.00% maintaining the same profile and 29.50% transitioning to the *Moderate Multiple-Goals* group.

At the overall sample level, profile consistency followed a similar pattern. Students in the *High Multiple-Goals* (60.00%) and *Mastery-Oriented* (57.10%) profiles demonstrated the highest levels of stability across domains. In contrast, students in the *Approach-Oriented* (43.40%) and especially the *Moderate Multiple-Goals* (23.10%) profiles were more likely to shift toward other motivational orientations, often toward more adaptive or complex profiles depending on the subject.

### 3.4. Associations Between Achievement Goal Profiles and Mathematics and Literacy Performance

In order to assess how achievement goal profiles were related to performance, two 2 (gender) × 4 (profiles) ANCOVAs (SES was included as a covariate) were conducted: one for mathematics and one for literacy performance as a dependent variable.

In mathematics, an initial ANCOVA including only gender and SES revealed a significant gender effect (*F*(1, 183) = 5.44, *p* = .021, *η*^2^*p* = 0.03) on mathematics performance, with boys (*M* = 14.22, *SE* = 0.33) outperforming girls (*M* = 13.16, *SE* = 0.31). However, when achievement goal profiles were added as a factor in a subsequent ANCOVA, the gender effect was no longer statistically significant (*F*(1, 183) = 3.87, *p* = .051, *η*^2^*p* = .02). A significant main effect of achievement goal profiles emerged (*F*(3, 181) = 4.79, *p* = .003, *η*^2^*p* = .08). Students in the *Mastery-Oriented* (*M* = 14.34, *SE* = 0.81) and *Approach-Oriented* (*M* = 14.69, *SE* = 0.42) profiles achieved the highest mathematics performance scores, compared to those in the *Moderate Multiple-Goals* (*M* = 11.93, *SE* = 0.63), and *High Multiple-Goals* (*M* = 13.48, *SE* = 0.32) profiles. However, post hoc comparisons (see Table 3) indicated that only students in the *Approach-Oriented* profile significantly outperformed those in the *Moderate Multiple-Goals* profile (*p* = .002). No other pairwise differences reached statistical significance (*p* > .05), including comparisons involving the *Mastery-Oriented* profile, the *High Multiple-Goals* profile, and the *Approach-Oriented* group. No significant *Gender × Profiles* interaction was found (*F*(3, 181) = 0.32, *p* = .810, *η*^2^*p* = .01).

In literacy, an initial ANCOVA including only gender and SES revealed a significant gender effect (*F*(1, 183) = 11.94, *p* < .001, *η*^2^*p* = .06), with girls (*M* = 13.71, *SE* = 0.45) outperforming boys (*M* = 11.37, *SE* = 0.42). This gender effect remained significant when achievement goal profiles were added to the model in a subsequent ANCOVA (*F*(1, 183) = 14.63, *p* < .001, *η*^2^*p* = .08), indicating a robust association between gender and literacy performance. A significant main effect of achievement goal profiles was also observed (*F*(3, 181) = 3.06, *p* = .030, *η*^2^*p* = .05). Students in the *Approach-Oriented* profile achieved the higher literacy scores (*M* = 14.07, *SE* = 0.71), compared to those in the *Mastery-Oriented* (*M* = 12.45, *SE* = 0.75), *Moderate Multiple-Goals* (*M* = 12.02, *SE* = 0.51), and *High Multiple-Goals* (*M* = 11.61, *SE* = 0.41) profiles. However, post hoc analyses (see Table 3) revealed that only the difference between the *Approach-Oriented* and *High Multiple-Goals* profiles was statistically significant (*p* = .019). No differences were found between the *Approach-Oriented* and *Mastery-Oriented* profiles (*p* = .709), nor between the *Approach-Oriented* and *Moderate Multiple-Goals* profiles (*p* = .122). Similarly, the *Mastery-Oriented* profile did not differ from either the *Moderate Multiple-Goals* or the *High Multiple-Goals* profiles (*p* > .05). No significant *Gender × Profiles* interaction was detected (*F*(3, 181) = 0.73, *p* = .538, *η*^2^*p* = .01).

## 4. Discussion

The present study aimed to identify achievement goal profiles among third-grade students using a person-centered approach and to examine their associations with academic performance in mathematics and literacy.

### 4.1. Achievement Goal Profiles Among Primary School Students

Using LPA, we identified the four expected motivational profiles across both academic domains: *Mastery-Oriented*, *Approach-Oriented*, *High Multiple-Goals*, and *Moderate Multiple-Goals*. These profiles are similar to those reported in earlier person-centered studies among elementary school students (e.g., Hornstra et al. 2017; Schwinger and Wild 2012). In that prior research, achievement goals in primary education have most often been examined within the trichotomous framework (i.e., mastery-approach, performance-approach, and performance-avoidance), partly due to concerns about the developmental relevance or clarity of mastery-avoidance goals for young children. By contrast, our study initially sought to apply the full 2 × 2 framework (Elliot and McGregor 2001), but performance-avoidance goals had to be excluded for psychometric reasons. As a result, our analyses did not fully align with this model. They also departed from the traditional trichotomous model, since our profiles retained mastery-avoidance goals alongside mastery- and performance-approach goals.

This configuration offers novel insight into the developmental role of mastery-avoidance in early schooling but also raises important questions. Although students in our sample appeared to clearly understand and endorse mastery-approach, performance-approach and mastery-avoidance goals, performance-avoidance goals seemed less meaningful or interpretable. One possible explanation lies in the type of standard each goal implies. Performance-avoidance goals involve a socially comparative and negatively framed benchmark (e.g., not wanting to be the worst), which assumes the presence of explicit rankings or normative evaluations. In contemporary primary classrooms, where traditional grades are often replaced by qualitative descriptors such as “*acquired*”, “*in progress*”, or “*not acquired*”, these forms of comparison may be less salient. Related evidence suggests that the presence of explicit normative evaluation can activate avoidance-oriented motives: for instance, anticipating a grade has been shown to increase the adoption of performance-avoidance goals, even when accompanied by formative comments (Pulfrey et al. 2011; see Koenka et al. 2021 for a meta-analysis). Large-scale survey data also indicate that performance-avoidance levels tend to rise with grade level, potentially reflecting the stronger emphasis on normative assessment practices in later schooling (U.S. Department of Education, Institute of Education Sciences 2017). In contrast, performance-approach goals (e.g., striving to be the best) may remain more accessible even in non-competitive environments, drawing on aspirational ideals that are familiar and motivating for many children. Likewise, mastery-avoidance goals focused on internal standards (e.g., fear of not understanding or forgetting), may be easier for young learners to grasp because they are frequently encouraged to monitor their learning progress. While these considerations are consistent with our interpretation, direct empirical evidence for this specific mechanism in early primary education is lacking, and future studies should investigate it explicitly, for example by examining how different classroom evaluation practices influence the accessibility of performance-avoidance goals.

### 4.2. Gender Differences

Beyond achievement goal profiles, this study explored the role of individual and contextual factors in relation to the adoption and stability of these profiles, with a particular focus on gender. Contrary to what is often reported in the literature (e.g., Schwinger et al. 2016; Zhang et al. 2016, 2023), girls were overrepresented in the *High Multiple-Goals* profile across both mathematics and literacy (60.20% and 50%, respectively). These findings suggest that by the end of the first cycle of primary school, girls may already internalize a broader and potentially demanding set of motivational standards, striving not only to learn and succeed but also to avoid failure and meet performance expectations. However, given the modest size of the effects and the absence of significant differences for some contrasts, these observations should be interpreted as indicative tendencies rather than stable gender-specific patterns.

This complex motivational pattern may reflect an early sensitivity to achievement pressure. Else-Quest et al. (2006) found that girls display higher levels of effortful control and perceptual sensitivity, making them more attuned to external expectations and potentially more prone to internalizing performance-related demands. In adolescence, this heightened sensitivity is often accompanied by increased anxiety, particularly in mathematics (Madjar et al. 2018), suggesting a developmental continuity between early motivational complexity and later emotional vulnerability.

In contrast, boys showed a more balanced distribution across profiles and were more likely to adopt the *Approach-Oriented* profile, particularly in literacy (25.29% vs. 9.18% for girls). A similar, though less pronounced, pattern was observed in mathematics (34.48% boys vs. 23.47% girls). This suggests that boys may be more likely to adopt motivational orientations emphasizing competence demonstration without the additional burden of avoidance-related concerns. Nonetheless, these differences were modest, and it would be premature to generalise beyond the present sample without replication.

Profile stability across academic domains also varied by gender. Among girls, the *High Multiple-Goals* profile showed the highest cross-domain consistency (64.40%), indicating a strong and persistent motivational signature. Conversely, a substantial proportion of girls classified as *Approach-Oriented* in mathematics were identified as *High Multiple-Goals* in literacy, suggesting a tendency to incorporate additional goals, potentially avoidance-related. It could be that the socially evaluative nature of the literacy domain is perceived as more demanding, especially by girls, who on average demonstrate higher achievement and are often considered to be “naturally” skilled in this subject (Abdullah et al. 2024; Bian et al. 2017; Eccles et al. 1993; Li et al. 2023). Boys, in contrast, showed relatively high profile stability in both the *Mastery-Oriented* and *Approach-Oriented* profiles. This may reflect a more compartmentalized motivational structure, with fewer shifts toward more complex or ambivalent motivational patterns.

Taking together, these findings reveal early gender differences in the structure and stability of achievement goal profiles. They underscore that girls are not uniformly mastery-oriented but may instead combine multiple and sometimes competing goals, especially in STEM (Science, Technology, Engineering, and Mathematics), where societal expectations and stereotype pressures may be more salient (Pintrich 2000; Repeykova et al. 2024; Spencer et al. 1999). This complexity may place girls at increased risk for overload or anxiety, especially in literacy where they outperform boys, and in mathematics where they perform less well than boys but may still face strong societal expectations to succeed. Evidence from older student populations further supports this interpretation: in a study of high-achieving adolescents (13–17 years), anxiety and educational track predicted achievement, but no gender differences were found in mathematics-related subjects, while girls consistently outperformed boys across a broad range of other subjects (Likhanov et al. 2024). Such findings suggest that gender differences in achievement may be domain-specific, and that heightened anxiety—rather than uniform performance gaps—could be a key factor underlying girls’ complex motivational patterns. Boys, on the other hand, tend to adopt simpler and more stable motivational orientations, centered on competence and performance.

These gendered pathways may be influenced by broader socialization processes, including parental expectations and behaviours. Prior research indicates that parental academic involvement can vary across domains and between genders, and that children may interpret such involvement differently depending on internalised stereotypes (Cvencek et al. 2011; Grolnick and Slowiaczek 1994; Pomerantz et al. 2007). Girls, for instance, may perceive high parental involvement in mathematics as pressure to overcome perceived deficits, leading to increased performance-approach or even avoidance goals. Boys, in contrast, might experience parental involvement in literacy as controlling or intrusive, thus undermining their intrinsic motivation in that domain. Given the modest effect sizes and non-significant differences for several gender contrasts in our study, these interpretations should be viewed as preliminary insights that point to directions for future research rather than firm conclusions.

This raises important questions for future research. One promising direction would be to explore how children’s perceptions of parental academic involvement relate to the formation of achievement goal profiles in a gender- and domain-specific manner. While prior studies have shown that supportive involvement fosters mastery-oriented goals, and controlling involvement predicts performance-oriented ones (Fan and Williams 2010; Gonzalez and Wolters 2006; Xiang et al. 2017), these effects have not been explored using person-centered approaches in primary school populations. Investigating how these perceptions interact with gendered academic stereotypes and domain-specific expectations could clarify the motivational mechanisms underpinning early gender gaps in achievement, particularly in mathematics and literacy.

### 4.3. Achievement Goal Profiles and Mathematics and Literacy Performance

Our second objective was to examine whether the identified motivational profiles were associated with differences in academic achievement in two core subjects of early education: mathematics and literacy. Results supported our hypothesis, though patterns differed slightly across domains.

In mathematics, students in the *Approach-Oriented* profile scored significantly higher than those in the *Moderate Multiple-Goals* profile, suggesting that a strong orientation toward both mastery- and performance-approach goals may offer a distinct advantage over a more ambivalent motivational pattern that includes moderate levels of mastery-avoidance. In literacy, the same *Approach-Oriented* group also significantly outperformed students in the *High Multiple-Goals* profile, who endorsed high levels of all goals, including mastery-avoidance.

These findings reinforce the idea that combining mastery- and performance-approach goals, in the absence of avoidance-related motivations, is particularly beneficial for academic achievement in elementary school. This pattern echoes previous research showing that such dual approach orientations are associated with greater engagement and higher academic outcomes (Harackiewicz et al. 2002a; Hornstra et al. 2017). Conversely, profiles characterized by avoidance motivations, even when accompanied by high approach goals, tend to be less adaptive, likely due to increased anxiety, concerns about failure, and the use of maladaptive strategies like self-handicapping, which ultimately harm academic success (Schwinger et al. 2022; Wirthwein et al. 2013).

Together, these findings suggest that even in early education, some students already endorse complex goal patterns that may hinder academic outcomes. By highlighting the relative benefits of *Approach-Oriented* profile and the potential risks associated with *Multiple-Goals* profiles, particularly those including mastery-avoidance goals, this study underscores the need for interventions that help young learners cultivate adaptive motivational orientations. However, it is important to note that the observed differences were small in magnitude and statistically significant only for some comparisons, suggesting that these findings should be interpreted as promising indications rather than robust prescriptions. Future research should continue to explore how these motivational profiles evolve over time and examine how they can be shaped through pedagogical practices, supportive teacher-student interactions, and family involvement. A growing body of evidence suggests that achievement goals are malleable and highly context-dependent (Ames 1992; Kaplan and Maehr 2007; Meece et al. 2006). For instance, the classroom goal structure or teacher discourse can influence whether students adopt mastery- or performance-oriented goals (Murayama and Elliot 2009). Moreover, experimental studies have shown that fostering a mastery focus can reduce stereotype threat and enhance academic outcomes (Canning et al. 2019; Good et al. 2003). These findings support the potential value of interventions aimed at reinforcing mastery- and performance-approach goals while minimizing avoidance motivations, in order to promote academic engagement and success in both mathematics and literacy.

### 4.4. Implications for Practice and Policy Makers

Although the associations between motivational profiles and achievement in our study were modest, and significant only in certain contrasts, they nevertheless point to concrete directions for practice and policy. One promising avenue is to normalise mistakes and frame them as integral to the learning process. Classrooms that promote a positive “error climate”—where errors are discussed openly and used as opportunities for growth—are associated with more adaptive motivational beliefs and higher engagement (e.g., Steuer and Dresel 2015; Soncini et al. 2022; Tulis et al. 2016). In mathematics education, the “productive failure” approach (Kapur 2008, 2014) exemplifies this principle: students first work on challenging problems without immediate instruction, encountering and analysing errors, before receiving targeted consolidation. Studies have shown that, under these conditions, students develop deeper conceptual understanding and are more likely to adopt mastery-oriented strategies (Kapur and Bielaczyc 2012). Embedding such practices in early primary classrooms could counteract tendencies toward mastery-avoidance by making struggle both safe and meaningful.

Beyond error climate, classroom-level recommendations align with broader research on goal structures, which shows that mastery-oriented climates—characterised by appropriately challenging tasks, opportunities for autonomy, cooperative learning, and recognition based on improvement—are associated with more adaptive achievement goals and better learning outcomes (e.g., Bardach et al. 2019; Kaplan and Maehr 2007; Meece et al. 2006). Designing such climates in a way that allows for both mastery and performance-approach goals, while minimising avoidance, may be especially beneficial.

Finally, because motivational beliefs develop through interactions both at school and at home, engaging families is crucial. Research has shown that process-focused parental feedback—praising effort, strategy use, and persistence—fosters incremental mindsets and adaptive goal orientations (Gonzalez and Wolters 2006; Pomerantz et al. 2007; Zong et al. 2018). Structured, low-stakes home activities in reading and mathematics, accompanied by guidance on avoiding ability labels or comparisons, can reinforce the classroom messages that learning is about growth rather than perfection.

### 4.5. Limitations and Future Directions

Several limitations of the present study should be acknowledged. First, the cross-sectional design prevents us from drawing causal conclusions regarding the relationships between gender, achievement goal profiles, and academic outcomes. Longitudinal research is needed to explore how motivational profiles evolve over time and to assess their long-term implications for academic trajectories. Second, although the sample of 185 third-grade students was sufficient to detect small to moderate effects, future studies would benefit from including larger and more diverse populations to strengthen the generalizability of the findings. The relatively small sample could have also affected results of the confirmatory factor analysis, for which the current sample is considered as just over the minimum recommended level (Kyriazos 2018). Third, the adaptation of existing instruments for use with young children posed notable psychometric challenges. While the study initially aimed to cover the full 2 × 2 framework of achievement goals, items assessing performance-avoidance were excluded due to low reliability. This may have constrained the identification of certain profiles through LPA. These challenges highlight the complexity of applying theoretically grounded models in early education settings and underscore the need for further instrument development and validation tailored to this developmental stage.

Finally, the study focused exclusively on self-reported motivational constructs and academic performance, without integrating other potentially influential factors such as classroom climate, teacher expectations, or peer dynamics. These contextual elements may interact with children’s goal orientations and contribute to shaping their academic engagement and outcomes. Future research could explore how achievement goal profiles are shaped not only by individual and school-level factors but also by family-related influences. In particular, examining the role of perceived parental academic involvement may provide valuable insights into the development of children’s motivation and performance. Investigating how parental academic involvement interacts with gender and motivational profiles could help identify protective or risk factors that contribute to academic inequalities from an early age.

## 5. Conclusions

This study provides new insights into the achievement goal profiles of third-grade students and their links to academic performance in mathematics and literacy, two foundational domains of learning. By using a person-centered approach, it reveals that adaptive profiles combining mastery- and performance-approach goals were associated with more favorable outcomes, whereas profiles involving multiple, often conflicting goals—particularly when avoidance motives were present—tended to be less beneficial. Moreover, the results tend to indicate gendered patterns in motivational development, with girls more often displaying complex multiple-goal profiles and boys more frequently represented in approach-oriented profiles. Interventions that help children cultivate adaptive goal orientations may be particularly effective in supporting academic engagement and reducing early achievement gaps.

In practical terms, our results point to the potential benefits of creating classroom environments that encourage mastery- and performance-approach goals while reducing avoidance tendencies, for example by fostering a positive error climate or embedding productive failure tasks. They also highlight the role of progress-focused feedback and perceived family engagement in reinforcing adaptive profiles. Although the observed effects were modest, implementing these approaches early and consistently could have meaningful cumulative benefits for students’ academic trajectories. Future work should test these strategies longitudinally to determine their long-term impact on learning and motivation and should also incorporate the perception of students and their parents to more fully capture the psychosocial mechanisms shaping achievement goal profiles.

## Figures and Tables

**Table 1:** Descriptive statistics and Pearson correlation coefficients for study variables.

Variable	*M*	*SD*	1	2	3	4	5	6	7	8	9	10
1. Gender (*0 = girl, 1 = boy*)												
2. SES	110.82	34.62	−.04									
3. Mathematics Performance	13.66	3.21	.15 *	.25 ***								
4. Mathematics Mastery-Approach Goal	4.51	0.70	−.06	−.03	.14							
5. Mathematics Mastery-Avoidance Goal	3.25	1.16	−.19 *	−.22 **	−.22 **	.13						
6. Mathematics Performance-Approach Goal	4.07	1.06	−.08	−.14	−.04	.49 ***	.05					
7. Literacy Performance	12.36	3.92	−.25 ***	.26 ***	.49 ***	.07	−.27 ***	−.007				
8. Literacy Mastery-Approach Goal	4.49	0.69	−.04	−.12	−.05	.45 **	.05	.48 ***	−.02			
9. Literacy Mastery-Avoidance Goal	3.33	1.21	−.20 *	−.14	−.19 **	.09	.58 ***	.06	−.22 **	.06		
10. Literacy Performance-Approach Goal	3.98	1.11	.002	−.17 *	−.04	.36 ***	.05	.75 ***	−.01	.56 ***	.05	

Note. * *p* < .05. ** *p* < .01. *** *p* < .001.

**Table 2:** Cross-tabulation of students’ achievement goal profiles in mathematics and literacy by gender (in %).

		Literacy Profiles			
Gender	Mathematics Profiles	*Mastery-Oriented*	*Approach-Oriented*	*Moderate Multiple-Goals*	*High Multiple-Goals*
Girls	*Mastery-Oriented*	**50.00%**	0.00%	50.00%	0.00%
	*Approach-Oriented*	13.0.%	**26.10%**	17.40%	43.50%
	*Moderate Multiple-Goals*	40.00%	0.00%	**50.00%**	10.00%
	*High Multiple-Goals*	3.40%	5.10%	27.10%	**64.40%**
Boys	*Mastery-Oriented*	**62.50%**	0.00%	37.50%	0.00%
	*Approach-Oriented*	0.00%	**56.70%**	13.30%	30.00%
	*Moderate Multiple-Goals*	38.50%	7.70%	**23.10%**	30.80%
	*High Multiple-Goals*	2.80%	11.10%	33.30%	**52.80%**
Total	*Mastery-Oriented*	**57.10%**	0.00%	42.90%	0.00%
	*Approach-Oriented*	5.70%	**43.40%**	15.10%	35.80%
	*Moderate Multiple-Goals*	39.10%	4.30%	**34.80%**	21.70%
	*High Multiple-Goals*	3.20%	7.40%	29.50%	**60.00%**

Note. Percentages reflect the proportion of students assigned to each literacy profile conditional on their mathematics profile, separated by gender and presented overall. Bold values indicate the percentages of students who were classified in the same profile in both mathematics and literacy.

**Table 3:** Descriptive statistics and group comparisons for mathematics and literacy performance according to achievement goal profiles.

Performance	Mastery-Oriented	Approach-Oriented	Moderate Multiple-Goals	High Multiple-Goals	Post hoc Comparisons
	*Mean (SD)*	*Mean (SD)*	*Mean (SD)*	*Mean (SD)*	Bonferroni
Math	14.34 (0.81)	14.69 (0.42)	11.93 (0.63)	13.48 (0.32)	**App > MM** **MO = MM = HM**
Literacy	12.45 (0.75)	14.07 (0.71)	12.02 (0.52)	11.61 (0.41)	**App > HM** **MO = MM = HM**

Note. MO = Mastery-Oriented; App = Approach-Oriented; MM = Moderate Multiple-Goals; HM = High Multiple-Goals. Group comparisons indicate statistically significant differences based on post hoc tests (*p* < .05). Greater than signs (>) indicate significantly higher performance, equals sign (=) indicates non-significant differences.

## Data Availability

The data and materials necessary to reproduce the analyses presented here are available on request from the corresponding author.

## Supplementary Materials

The following supporting information can be downloaded at: https://www.mdpi.com/article/10.3390/jintelligence13090108/s1. Table S1: Supplementary table of latent profile solutions for mathematics and literacy achievement goals. Figure S1: Scatterplots illustrating the associations between mastery-avoidance goals and academic performance in mathematics (Panel A) and literacy (Panel B), separately for girls and boys. MATH: Mathematics performance; LITERACY: Literacy performance; M_Mav: Mathematics Mastery-Avoidance Goals; L_Mav: Literacy Mastery-Avoidance Goals. Shaded areas represent 95% confidence intervals.

## Abbreviations

The following abbreviations are used in this manuscript:

LPA	Latent Profile Analysis
SPI	Social Position Index
SES	Socioeconomic Status
ANCOVA	Analysis of Covariance
STEM	Science, Technology, Engineering, and Mathematics
