# Supplementary material for: Achievement Goal Profiles and Academic Performance in Mathematics and Literacy: A Person-Centered Approach in Third Grade Students
Source: J Intell. 2025 Aug 27;13(9):108. doi: 10.3390/jintelligence13090108 (PMC12470418; doi:10.3390/jintelligence13090108)
Supplement: Supplementary file 1 [file jintelligence-13-00108-s001.zip › jintelligence-3798250-supplementary.pdf]

Supplementary Materials

Some analyses required more detailed reporting to ensure full transparency and interpretability. Therefore, we provide this Supplementary Materials to present extended results that complement the main findings.

1. Descriptive Statistics

1.1. Gender Differences Across All Study Variables

We examined gender differences across all study variables using Pearson correlations (with gender coded as 0 = girl, 1 = boy). Consistent with the main findings, significant gender differences emerged in students' academic performance. Boys (M = 14.18, SD = 3.02) outperformed girls in mathematics (M = 13.19, SD = 3.31; Cohen's d = .31, p = .046), whereas girls (M = 13.28, SD = 3.73) outperformed boys in literacy (M = 11.32, SD = 3.89; Cohen's d = .52, p < .001).

Beyond academic performance, gender differences were also observed in students' achievement goals. Girls reported higher levels of mastery-avoidance goals than boys in both subjects: mathematics (Mgirls = 3.45, SEgirls = 0.11 vs. Mboys = 3.01, SEboys = 0.12; Cohen's d = .41, p = .006) and literacy (Mgirls = 3.56, SEgirls = 0.12 vs. Mboys = 3.10, SEboys = 0.13; Cohen's d = .41, p = .006). This suggests that girls were more likely to focus on avoiding misunderstanding or failure to master a task, a tendency observed independently in mathematics and literacy. In contrast, no significant gender differences were found for mastery-approach (in math: Cohen's d = .11, p = .452; in literacy: Cohen's d = .09, p = .571) or performance-approach (in math: Cohen's d = .17, p = .246; in literacy: Cohen's d = .00, p = . 939) goals in either domain. These results indicate that while boys and girls generally endorsed achievement goals to a similar degree, they differed in their susceptibility to avoidance-oriented motivational tendencies.

1.2. Associations Between Mastery-Avoidance Goals and Academic Performance

To further illustrate these effects, we examined the associations between mastery-avoidance goals and domain-specific performance using Pearson correlations. As reported in the main text, mastery-avoidance was negatively correlated with mathematics (r = –.22, p = .003) and literacy (r = –.22, p = .003) performance.

Figure S1 displays scatterplots of these associations, separated by gender (0 = girls, 1 = boys). In both mathematics and literacy, higher endorsement of mastery-avoidance goals was associated with lower achievement, confirming the negative correlations. The regression lines show that this trend was evident for both girls and boys, although the slopes appear somewhat steeper for boys in literacy. This suggests that while girls reported higher overall mastery-avoidance tendencies, the detrimental association with performance may be particularly pronounced for boys in the literacy domain.

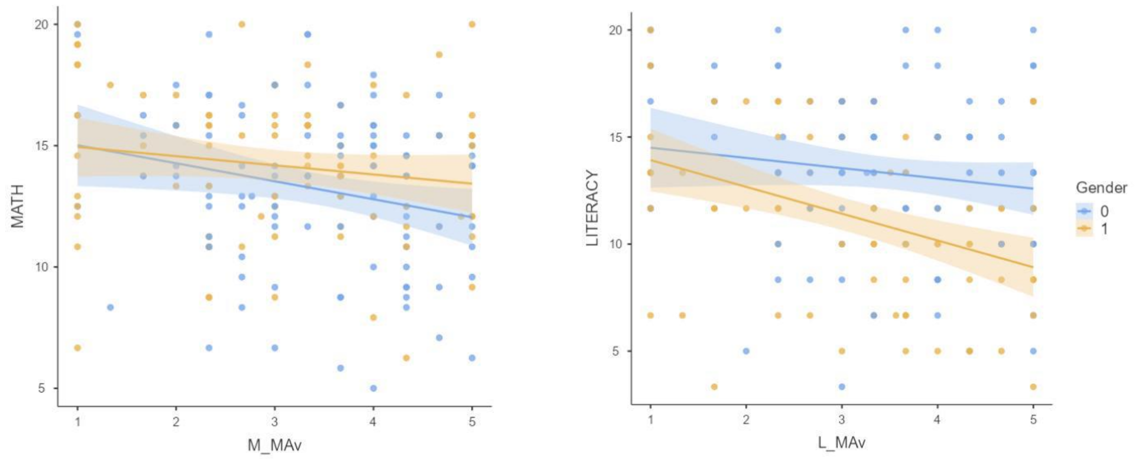

A **B**

Figure S1. Scatterplots illustrating the associations between mastery-avoidance goals and academic performance in mathematics (Panel A) and literacy (Panel B), separately for girls and boys. MATH: Mathematics performance; LITERACY: Literacy performance; M_Mav: Mathematics Mastery-Avoidance Goals; L_Mav: Literacy Mastery-Avoidance Goals. Shaded areas represent 95% confidence intervals.

1.3. Associations Between Achievement Goals and Academic Performance

In mathematics, a significant negative association was observed between mastery-avoidance goals and performance ($r = -.22$, $p = .003$), indicating that students who were motivated by a desire to avoid misunderstanding or failing to fully master the material tended to perform more poorly. Neither mastery-approach ($r = .14$, p = .062) nor performance-approach ($r = -.04$, p = .560) goals were significantly related to mathematics performance.

A similar pattern emerged in literacy. Again, only mastery-avoidance goals showed a significant negative association with performance ($r = -.22$, $p = .003$), reinforcing the idea that avoidance-driven motivations may hinder academic success. As in mathematics, no significant associations were found between either mastery-approach ($r = -.02$, $p = .744$) or performance-approach ($r = -.01$, p = .886) goals and literacy performance.

1.4. Cross-Domain Relations Between Achievement Goals

We also examined the interrelations among achievement goals within each academic domain. In both mathematics and literacy, mastery-approach goals were significantly correlated with performance-approach goals (mathematics: $r = .49$, $p < .001$; literacy: $r = .56$, $p < .001$), likely reflecting their shared positive valence and focus on success-oriented striving.

In contrast, no significant associations were found between mastery-approach and mastery-avoidance goals (mathematics: $r = .13$, $p = .081$; literacy: $r = .06$, $p = .411$), nor between mastery-avoidance and performance-approach goals (mathematics: $r = .05$, $p = .466$; literacy: $r = .05$, $p = .523$).

2. Identification of Achievement Goals Profiles

To identify achievement goal profiles separately for mathematics and literacy, we used Latent Profile Analysis (LPA), a person-centered statistical approach that classifies individuals into subgroups based on shared patterns of achievement goal orientations (Hornstra et al. 2017; Jansen in de Wal et al. 2015; Wormington & Linnenbrink-Garcia 2017). LPA determines the optimal number of latent profiles that best describe the data, providing a nuanced understanding of students' motivational structures and the potential combined effects of multiple goals (Muthén & Muthén 2000; Pastor et al. 2007). Achievement goal scores were used as indicators of a latent categorical variable, representing unobserved subgroups within the data. Separate LPA models were estimated for mathematics and literacy, testing solutions ranging from one to four profiles to determine the best-fitting model.

For LPA models, model fit was evaluated using multiple statistical indices (see Table S1), including Akaike's Information Criterion (AIC; Akaike 1998), Bayesian Information Criterion (BIC; Schwarz 1978), and the sample-size adjusted BIC (SABIC; Sclove 1987), with lower values indicating better fit. To compare nested models, we relied on the Bootstrap Likelihood Ratio Test (BLRT; Nylund et al. 2007), where a significant p-value indicated that the k-profile solution provided a significantly better fit than a solution with k–1 profile. The entropy value, which reflects classification accuracy, was not used as the sole decision criterion because it is sensitive to the number of profiles, which often increases with more profiles.

Table S1

Supplementary Table of Latent Profile Solutions for Mathematics and Literacy Achievement Goals

	Mathematics						Literacy					
$N_{Classes}$	*LogL*	*AIC*	*BIC*	*SABIC*	*BLRT*	*Entropy*	*LogL*	*AIC*	*BIC*	*SABIC*	*BLRT*	*Entropy*
2	-699.68	1419.37	1451.57	1419.90	0.010	0.95	-711.86	1443.71	1475.92	1444.24	0.010	0.87
3	-692.76	1413.52	1458.61	1414.26	0.079	0.77	-700.81	1429.63	1474.71	1430.37	0.010	0.79
**4**	**-668.81**	**1379.63**	**1447.26**	**1380.74**	**0.010**	**0.79**	**-680.25**	**1396.50**	**1454.46**	**1397.45**	**0.010**	**0.83**

Note. LogL = Log Likelihood; AIC = Akaike's Information Criterion; BIC = Bayesian Information Criterion; SABIC = sample-size adjusted BIC; BLRT = Bootstrap Likelihood Ratio Test. For the BLRT, reported numbers are p-values. Bold values represent the final model chosen. .

We tested profile solutions ranging from one to four classes and chose to limit our analysis to four-profile solutions, in line with previous research indicating that, within a trichotomous achievement goal framework, the number of observed profiles rarely exceeds four. For both the mathematics and literacy domains, the four-profile solution provided the best fit and most meaningful differentiation of student's achievement goal patterns. This decision was further supported by theoretical considerations related to the plausibility and interpretability of the profiles. Each latent profile was labeled based on its mean achievement goal scores and in reference to established typologies in the existing literature.

2.1. Mathematics Achievement Goal Profiles

The first math profile, labeled *Mastery-Oriented* profile, comprised 7.57% of students. It was characterized by high mastery-approach (*M* = 4.33, *SE* = 0.29) and mastery-avoidance goals (*M* = 3.57, *SE* = 0.43), with low performance-approach goals (*M* = 2.28, *SE* = 0.40). This profile was the least common and displayed a relatively even gender distribution (6.12% girls, 9.20% boys).

The second profile, *Approach-Oriented* profile, comprising 28.65% of students, combined high mastery-approach (*M* = 4.82, *SE* = 0.06) and performance-approach goals (*M* = 4.56, *SE* = 0.13), with low mastery-avoidance goals (*M* = 2.04, *SE* = 0.16). It appeared more frequently among boys (34.48%) than girls (23.47%).

The third profile was the *Moderate Multiple-Goals* profile. It included 12.43% of students and reported moderate levels of mastery-approach (*M* = 2.94, *SE* = 0.30), mastery-avoidance (*M* = 3.08, *SE* = 0.31), and performance-approach goals (*M* = 3.07, *SE* = 0.39). This group was evenly distributed by gender (10.20% girls, 14.94% boys).

The last and largest profile was the *High Multiple-Goals* profile with 51.35% of students. It was marked by high mastery-approach (*M* = 4.75, *SE* = 0.06), mastery-avoidance (*M* = 3.92, *SE* = 0.15), and performance-approach goals (*M* = 4.39, *SE* = 0.13). This profile was most common among girls (60.20%) but also widely represented among boys (41.38%).

2.2. Literacy Achievement Goal Profiles

For literacy, the first profile was also identified as a *Mastery-Oriented* profile and represented 12.43% of students. It was characterized by high mastery-approach (*M* = 3.68, *SE* = 0.27), moderate mastery-avoidance goals (*M* = 2.91, *SE* = 0.32), and low performance-approach goals (*M* = 1.78, *SE* = 0.20). Although this was the least frequent profile in both domains, it was nearly twice as common in literacy (12.43%) as in mathematics (7.57%). As observed in math, this profile displayed a balanced gender distribution (12.24% girls, 12.64% boys).

The second profile, *Approach-Oriented* profile, was identified in 16.76% of students. It included high levels of mastery-approach (*M* = 4.88, *SE* = 0.04) and performance-approach goals (*M* = 4.84, *SE* = 0.06), alongside low mastery-avoidance goals (*M* = 1.70, *SE* = 0.14). This profile was less common in literacy (16.76%) than in mathematics (28.65%). A gender difference was again observed, with boys (25.29%) being more likely to belong to this profile than girls (9.18%). Notably, this gender gap was more pronounced in literacy than in mathematics (25.29% vs. 9.18% in literacy, compared to 34.48% vs. 23.47% in math).

The third profile, *Moderate Multiple-Goals* profile, included 27.03% of students. It was characterized by moderate scores across all three goals orientation: mastery-approach (*M* = 4.15, *SE* = 0.17), mastery-avoidance (*M* = 3.44, *SE* = 0.15), and performance-approach goals (*M* = 3.29, *SE* = 0.16). This profile was considerably more common in literacy (27.03%) than in mathematics (12.43%). Girls (28.57%) and boys (25.29%) were similarly represented, and both genders adopted this profile more frequently in literacy than in math (10.20% girls, 14.94% boys in math).

The final and most prevalent profile, *High Multiple-Goals* profile, encompassed 43.78% of students. As in mathematics, this profile was defined by high levels of all three goals: mastery-approach (*M* = 4.77, *SE* = 0.05), mastery-avoidance (*M* = 4.02, *SE* = 0.13), and performance-approach (*M* = 4.67, *SE* = 0.07). It was the most frequent in both

domains, representing 51.35% of students in mathematics and 43.78% in literacy. Girls were again more likely to endorse this profile (50%) than boys (36.78%), confirming the pattern observed in mathematics. However, while the gender difference remained in favor of girls in both domains, it was more pronounced in mathematics (+18.82 percentage points: 60.20% girls vs. 41.38% boys) than in literacy (+13.22 points: 50% girls vs. 36.78% boys).

References

Akaike, Hirotugu. 1998. A New Look at the Statistical Model Identification. In *Selected Papers of Hirotugu Akaike*. Edited by Emanuel Parzen, Kunio Tanabe, and Genshiro Kitagawa. New York: Springer Series in Statistics, pp. 215-222. [CrossRef]

Hornstra, Lisette, Marieke Majoor, and Thea Peetsma. 2017. Achievement Goal Profiles and Developments in Effort and Achievement in Upper Elementary School. *British Journal of Educational Psychology* 87: 606–29. [CrossRef] [PubMed]

Jansen in de Wal, Joost, Lisette Hornstra, Frans J. Prins, Thea Peetsma, and Ineke van der Veen. 2015. The Prevalence, Development and Domain Specificity of Elementary School Students' Achievement Goal Profiles. *Educational Psychology* 36: 1303–22. [CrossRef]

Muthén, B., and L. K. Muthén. 2000. Integrating Person-Centered and Variable-Centered Analyses: Growth Mixture Modeling with Latent Trajectory Classes. *Alcoholism, Clinical and Experimental Research* 24: 882–91. [CrossRef] [PubMed]

Nylund, Karen L., Tihomir Asparouhov, and Bengt O. Muthén. 2007. Deciding on the Number of Classes in Latent Class Analysis and Growth Mixture Modeling: A Monte Carlo Simulation Study. *Structural Equation Modeling* 14: 535–69. [CrossRef]

Pastor, Dena A., Kenneth E. Barron, B. J. Miller, and Susan L. Davis. 2007. A Latent Profile Analysis of College Students' Achievement Goal Orientation. *Contemporary Educational Psychology* 32: 8–47. [CrossRef]

Schwarz, Gideon. 1978. Estimating the Dimension of a Model. *The Annals of Statistics* 6: 461–64. [CrossRef]

Sclove, Stanley L. 1987. Application of Model-Selection Criteria to Some Problems in Multivariate Analysis. *Psychometrika* 52: 333–43. [CrossRef]

Wormington, Stephanie Virgine, and Lisa Linnenbrink-Garcia. 2017. A New Look at Multiple Goal Pursuit: The Promise of a Person-Centered Approach. *Educational Psychology Review* 29: 407–45. [CrossRef]
